# Discriminant canonical analysis as a tool for genotype traceability testing based on turkey meat and carcass traits

**DOI:** 10.3389/fvets.2024.1326519

**Published:** 2024-02-15

**Authors:** José Ignacio Salgado Pardo, Antonio González Ariza, Francisco Javier Navas González, José Manuel León Jurado, Esther Díaz Ruiz, Juan Vicente Delgado Bermejo, María Esperanza Camacho Vallejo

**Affiliations:** ^1^Department of Genetics, Faculty of Veterinary Sciences, University of Córdoba, Córdoba, Spain; ^2^Agropecuary Provincial Centre, Diputación Provincial de Córdoba, Córdoba, Spain; ^3^Department of Agriculture and Ecological Husbandry, Area of Agriculture and Environment, Andalusian Institute of Agricultural and Fisheries Research and Training (IFAPA), Alameda del Obispo, Córdoba, Spain

**Keywords:** breed traceability, weight-related traits, histological properties, sexual dimorphism, color-related traits, pH, gross nutrient, water-holding capacity

## Abstract

The present study aims to develop a statistical tool for turkey breed traceability testing based on meat and carcass quality characteristics. To this end, a comprehensive meta-analysis was performed, collecting data from a total of 75 studies approaching meat and carcass attributes of 37 turkey strains and landraces since the late 1960s. A total of 22 meat and carcass traits were considered variables, grouped in the following clusters: carcass dressing traits, muscle fiber properties, pH, colorimetry, water-capacity traits, texture-related attributes, and nutritional composition of the meat. Once the multicollinearity analysis allowed the deletion of redundant variables, cold carcass weight, slaughter weight, muscle fiber diameter, sex-female, carcass/piece weight, meat redness, ashes, pH24, meat lightness, moisture, fat, and water-holding capacity showed explanatory properties in the discriminating analysis (*p* < 0.05). In addition, strong positive and negative correlations were found among those variables studied. Carcass traits were positively associated, particularly slaughter weight and cold carcass weight (+0.561). Among meat physical traits, pH showed positive correlations with drip loss (+0.490) and pH24 (+0.327), and water-holding capacity was positively associated with cholesterol (+0.434) and negatively associated with collagen (−0.398). According to nutritional traits, fat and ash showed a strong correlation (+0.595), and both were negatively associated with moisture (−0.375 and −0.498, respectively). Strong negative correlations were found as well between meat protein and fat (−0.460) and between collagen and cholesterol (−0.654). Finally, the Mahalanobis distance suggested a clustering pattern based on meat and carcass characteristics that report information about interbreeding and variety proximity. This study establishes a departure point in the development of a tool for breed traceability guaranteeing aimed at enhancing distinguished, local breed-based turkey meat.

## 1 Introduction

The global meat industry has accomplished the goal of providing protein, and meat prices are currently at historic lows (1). Poultry emerges as the meat type with the most efficient production among the current livestock in terms of the use of resources and supply of protein (2), and turkey emerges second in terms of the largest contributor to global poultry meat production (3). Turkey has undergone great selection pressure to target desirable traits such as fast growth and high slaughter weight, which has led to a doubling of its production between 1970 and 2008 (3). However, its negative impact on meat quality (4–6), together with a bad image of their low-sustainable housing systems and welfare conditions, has led to the apparition of alternative, free-range systems using slow-growing strains (1). Provided that the loss of meat quality originates from the growth curve and its physiology, slow-growing strains seem to offer a differential quality product (4). These unconventional systems arise to meet the demand for higher quality meat production that is both sustainable and ethical (1, 7, 8). This trend is exemplified by the ‘Traditional Farmfresh Turkey’ labeling developed in the United Kingdom to distinguish traditional farming turkey meat from commercial products (9).

Those unconventional systems are mainly based on light, slow-growing landraces. The most widespread genotypes involved are heritage breeds such as the Royal Palm (10), Narragansett (11), or Beltsville Small White (12), and other light hybrids from the commercial industry (13, 14). However, those alternative systems represent a great growth opportunity for indigenous, locally adapted genotypes (15). In this respect, native turkeys are more suitable for free-ranging breeding than industrial strains. In harsh and disease-prone environments, a lack of performance and adaptation has been reported in imported genotypes (2). On the other hand, local breeds offer great heat tolerance and immunological competence (16) while preserving ancestral behaviors such as constant food-seeking or anti-predator conduct (16–20).

**Figure 1:**
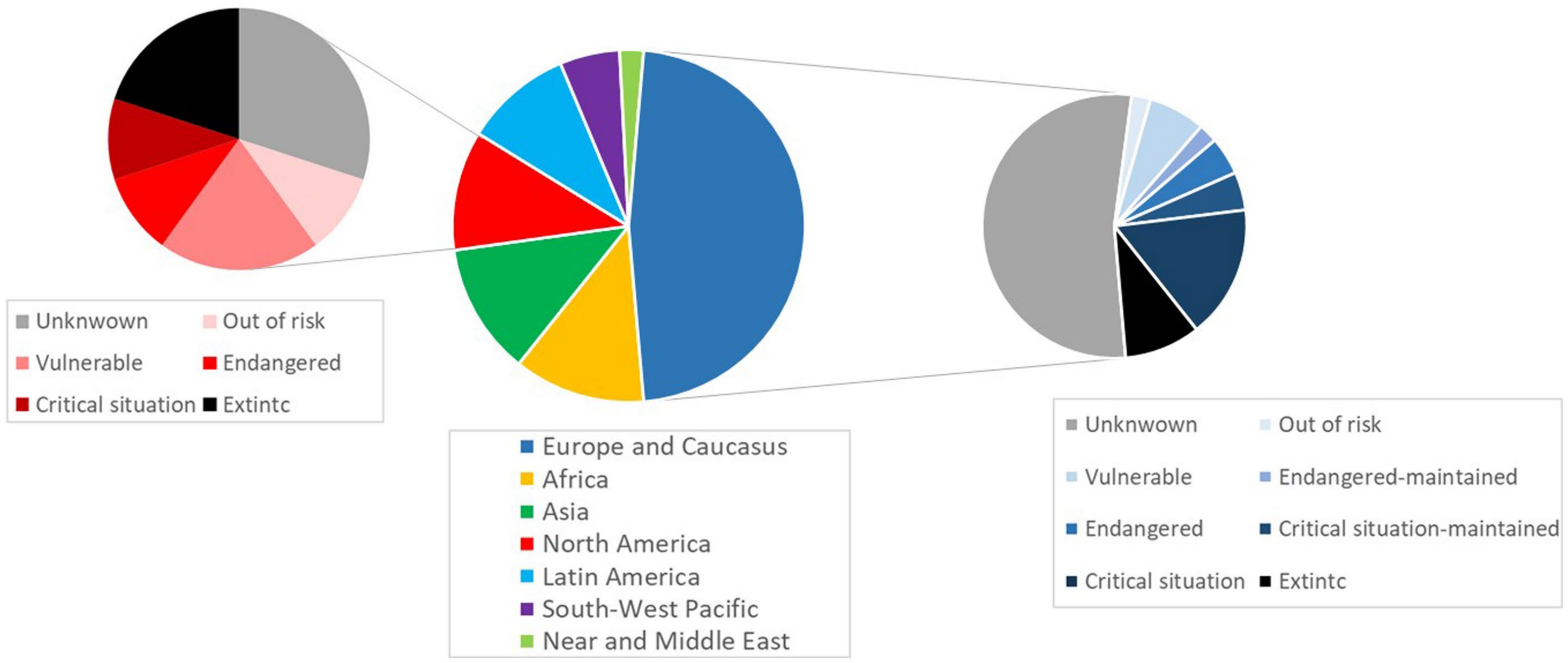
Distribution of worldwide turkey breeds and population status in North America and Europe. Source: Domestic Animal Diversity Information System (DAD-IS) (9).

In addition to their greater suitability for alternative systems, indigenous genotypes are preferred to industrial products by both rural and urban consumers (16, 21). For example, in China, native chicken is preferred to standard broilers because of its better taste and traits, which are well adapted to Chinese cuisine (22). A similar case is found in Italy, where the carcasses from Italian local turkey breeds suit the traditional Italian cuisine (23). These products represent distinguished, gourmet items that are usually associated with specific events, such as traditional festivities (24). In the United States, the consumption of their ‘heritage’ turkey breeds is linked with Thanksgiving, a national holiday (24). In Mexico, the indigenous domestic genotype known as ‘Guajolote’ is highly valued as a ceremonial food, consumed during family festivities, mainly in December (20). In this respect, enhancing the consumption of local breeds’ products could be a crucial strategy to preserve them (25). Most of the native poultry genotypes are threatened due to the rise of highly selected strains in the 20th century, via displacement and genetic erosion (2, 26). This is evidenced by the fact that 6.59% of turkey breeds have undergone extinction, and the status of 70.32% of the populations is still unknown (27). Furthermore, there are no data available about the endangered level of turkey populations worldwide except for some populations from Europe and North America, which are shown in Figure 1.

However, due to the use of higher land proportions and the lower feed efficiency of these alternative systems, those high-quality products, derived from local breeds, must command higher prices to achieve profitability (1). In addition, informed and conscious consumers must be willing to pay the extra price that these premium products, obtained from local breeds, are worth (16, 21, 22). In this respect, breed traceability is a crucial issue for ensuring the origin of traditional and regional foods (28). For example, when marketing carcasses and cuts from local breeds, fraud could occur through crossbreeding or using breeds other than those specified (29). Attempting to define a breed traceability tool, genomic and proteomic approaches have been proposed. However, its technical complexity and high price make molecular traceability unviable for small and low-income productions (28), in which native breeds are usually reared. On the other hand, when fewer resources are available, the status of a native genotype can be approached through phenotypes, since phenotypic traits are a direct consequence of the genotype (24). This approach has been followed in studies comparing meat and carcass quality attributes between turkey breeds and varieties (4–8, 13, 30, 31) and their crossbreeds (32–34).

A recent meta-analysis has proved that meat and carcass quality traits stand as explanatory variables in a discriminant canonical analysis (DCA) to describe clustering patterns among turkey genotypes (35). Authors suggested that, from the cornerstone established in the study, the discriminant function cross-validation analysis could be implemented as a breed traceability tool, which could mean great industrial applications. However, the study included just a few well-differentiated populations and did not deepen the correlation among traits. Describing the correlation of traits is crucial to increasing the efficiency of resource allocation in the method since the reduction of measures is possible by selecting highly explanatory and correlated variables. Thus, correlations between the most common meat and carcass traits have been reported in several poultry species (4, 36–38).

Hence, the present study aims to develop a statistical tool to perform a breed traceability test based on meat and carcass quality attributes. To achieve this goal, a study of the traits acting as differentiating patterns across turkey landraces and varieties within genotypes, together with their correlations, is conducted. Hence, the present study might present a discriminant canonical feature for breed traceability of traditional meat products based on local breeds, as well as to detect breed introgression and hybridization of those genotypes. This tool could be particularly useful for indigenous genotypes and could be employed as a guarantee of their distinguished, breed-based products. Due to the low cost and simplicity of these phenotypical measurements by selecting highly explanatory traits correlated with other significant ones, this tool is particularly suited to low-income and scarce resource systems that are characteristics of local breeds.

## 2 Materials and methods

### 2.1 Decision of the systematic review approach

Preferred Reporting Items for Systematic Reviews and Meta-Analyses (PRISMA) guidelines were designed for the healthcare field and do not fit properly in livestock research (39, 40). Furthermore, strict adherence to the PRISMA guidelines failed to detect any alterations in the journal’s level of recommendation and endorsement (41) and exhibited restricted suitability in the context of reviews concerning conservation and environmental management. Instead, the methodology selected in the present study was that described by McLean and Navas Gonzalez (42), which has been reported as an efficient methodology in previous studies (18, 39, 43).

### 2.2 Data collection

Data collection was executed as previously reported in the literature (18, 39, 43). To this end, the repositories www.scholar.google.es and www.sciencedirect.com (accessed on November 2021) were employed. Other platforms not including data extraction tools for analysis, such as www.ncbi.nlm.gov/pubmed or www.webofscience.com/wos/woscc/basic-search, were excluded, as suggested in the bibliography (18, 43). Restricted documents were accessed through the library service of the University of Córdoba (Córdoba, Spain). Keywords included in the document collection were “meat/carcass quality” and “meat/carcass traits” followed by “turkey”, “*Meleagris gallopavo*”, and other semantically related terms (18, 44). A total of 75 documents published in English from 1968 to 2021 were found and included in this research.

Documents were recorded in a database where observations were individually registered considering the turkey landrace. In this respect, a total of 37 varieties comprising 9 turkey landraces and well-established populations were obtained and are shown in Table 1. Table 1 also shows the reference from which each variety was collected. The carcass cuts used in the present study were the following: carcass reminder, breast, complete leg, thigh, drumstick, wings, head, neck, feet, shank, back, heart, liver, giblets, kidney, lungs, spleen, pancreas, gallbladder, proventriculus, gizzard (full and empty), stomach, complete intestine, small intestine, cecum, abdominal fat, fat pad, ovary, oviduct, feathers, skin, feather plus skin, blood, and waste. Moreover, the meat and carcass quality traits included in the analysis were as follows: carcass/piece weight, carcass/piece yield, cold carcass weight, slaughter weight, muscle fiber diameter, pH, pH 24 h, L* meat, a* meat, b* meat, drip loss, water-holding capacity, cooking loss, shear force, springiness, fragmentation index, moisture, protein, fat, ash, collagen, and cholesterol. To avoid possible errors when encountering different units of measurement in literature, all units were converted to the most frequently found units across papers. Specific methodologies used in every study for variable determination were not registered as the analysis methods employed were standardized to be accepted in research procedures. This determination is based on the fact that, although distinct techniques may cause differences, as they are standardized methods, these differences may be negligible (18).

**Table 1:** Landraces, varieties, and references where they were collected.

Breed	Variety or strain	Cites
Beltsville Small White	–	(45–49)
Commercial	AVIAGEN	(50)
	Broad breasted bronze	(46, 51)
	Bronze turkey strain	(52)
	BUT	(53–55)
	BUT 6	(31, 56)
	BUT 9	(57–61)
	BUT big 6	(8, 62–68)
	BUT large white	(69)
	Female line	(70)
	Hybrid 1000	(71)
	Hybrid converter	(31, 72, 73)
	Hybrid grade marker	(74)
	Hybrid Optima	(75)
	Kelly broad breasted bronze	(8)
	Kelly super mini	(8)
	Kelly Wrolstad	(8)
	Large white	(76)
	Male line	(70)
	Nandanam	(77)
	Nicholas	(56, 69, 78–82)
	Nicholas 500	(83)
	Nicholas 700	(31, 84)
	Nicholas BUT	(85)
	Nicholas large white	(86)
	Unspecified	(6, 72, 87–103)
	White turkey	(104)
	Williams	(56)
Local Egyptian	–	(105)
Local Lebanese	–	(60)
Local Nigerian	White plumage	(106)
	Black plumage	(106)
	Unspecified	(107, 108)
North Caucasian bronze	–	(109)
Turkish bronze	–	(104)
Wild turkey	*Meleagris gallopavo silvestris*	(109, 110)
Unspecified	–	(111–115)

### 2.3 Data analysis

#### 2.3.1 Normality and Bayesian ANOVA tests

To discard alterations of the normality assumption, the Shapiro–Francia W’ test was performed. This test was chosen because of the number of observations collected, which ranged from 50 to 2,500. After obtaining both normally and non-normally distributed variables, a Bayesian ANOVA was used to analyze differences between turkey varieties and strains. The results from the Bayesian ANOVA test reported medians to significantly differ in the majority of possibilities. However, the following variables did not report significant differences: pH (*F* = 0.747, *p*v = 0.674), a* meat (*F* = 0.388, *p*v = 0.960), b* meat (*F* = 1.718, *p*v = 0.101), drip loss (*F* = 0.487, *p*v = 0.848), cooking loss (*F* = 1.849, *p*v = 0.083), shear force (*F* = 0.248, *p*v = 0.983), and collagen (*F* = 35.764, *p*v = 0.105). Therefore, the presence of differences in these variables across the turkey varieties justified the implementation of a DCA.

#### 2.3.2 Multicollinearity preliminary testing

To discard linear relationships across predictors and guarantee the variable’s independence, the multicollinearity analyses were run before the statistical analyses *per se*. The objective of these analyses were to detect noise or redundancy issues in the variables before data manipulation, as the exclusion of unnecessary variables avoids a possible overinflation of the variance’s explanatory potential (39). The variance inflation factor (VIF) is employed as an indicator of multicollinearity, and values above 5 are not recommended (116). VIF is calculated with the following formula:


$$\mathrm{VIF}=\frac{1}{1-R^{2}},$$

where *R*^2^ represents the coefficient of determination of the regression equation and tolerance (1 – *R*^2^) reflects the degree of variability in a specific independent variable that is not explained by the rest, whose recommended values are under 0.20 (117). To perform the multicollinearity test, the multicollinearity statistics routine of the describing data package of XLSTAT software (Addinsoft Pearson Edition 2014, Addinsoft, Paris, France) was employed.

#### 2.3.3 Discriminant canonical analysis

To perform the statistical analysis, turkey landraces and varieties were considered the independent variables to perform the DCA, and the 22 meat and carcass parameters mentioned before were employed as explanatory dependent variables. For each carcass or carcass piece analyzed, the sex of the turkey breed from which it was obtained was included and used as a labeling classification criteria to determine the variability of quality-associated attributes between and within classification clusters and to establish, identify, and outline groupings (118).

A series of discriminant functions were obtained from the statistical analysis and enabled the definition of the clustering patterns described by the sample through a linear combination of meat and carcass quality-related attributes. For the selection of variables, regularized forward stepwise multinomial logistic regression algorithms were used (15). Instead of considering group sizes to be equal, priors were regularized following the group sizes computed from the prior probability option in SPSS version 26.0 software (IBM, Armonk, NY, United States), which prevents groups with different sample sizes from influencing classification quality (119).

#### 2.3.4 DCA efficiency and analysis model reliability

To determine variables significantly contributing to the discriminant function, Wilks’ lambda test was used, as described by González Ariza (118). Values under 0.05 or Wilks’ lambda values can be accepted (120), even though ideal values tend to 0.

For evaluating the assumption of equal covariance matrices in cases of unequal sample sizes, Pillai’s trace criterion is the only acceptable test (121) and was run using the multivariate routine of the general linear model package of the software SPSS, version 26.0. Statistical differences in the dependent variables across the levels of independent variables are suggested to be accepted when significance is under 0.05 (118).

#### 2.3.5 Correlation matrix

A correlation matrix among meat and carcass attributes obtained from the DCA was depicted in a graphical representation. To achieve this goal, a heat map was built through the web server Heatmapper (accessed on 18th April 2023).^1^ This analysis provides insights into what meat and carcass quality-related traits show a higher correlation between them. With these insights, the knowledge generated in the correlation matrix will allow an optimization of the data collection process.

#### 2.3.6 Variable dimensionality reduction

Overall, variables were narrowed down to the few significant variables that most contributed to the different variations in the different types of birds, which was done through a preliminary principal component analysis (PCA) according to the bibliography (118).

#### 2.3.7 Canonical coefficients and loading interpretation and spatial representation

A discriminant function analysis was employed to determine the degree of assignment of a carcass or a primary cut within its group (which was defined by the turkey variety). Hence, variables exhibiting a discriminant loading of ≥|0.40| were considered to be substantially discriminant, according to the literature (118). In this respect, the discriminant ability was determined considering the absolute coefficients for each particular variable within a series (122). Consecutively, the squared Mahalanobis distance were calculated following this formula:


$$D_{ij}^{2}=\left(\bar{Y_{i}}-\bar{Y_{j}}\right)\;COV^{-1}\left(\bar{Y_{i}}-\bar{Y_{j}}\right),$$

where D^2^_ij_ represents the distance between population *i* and *j*; *Ȳ*_i_ and *Ȳ*_j_ represent the means of variable x in the *i*th and *j*th populations, respectively; and COV^−1^ represents the inverse of the covariance matrix of measured variable x (120).

The clustering patterns of the observations were visually represented through the squared Mahalanobis distance, which can be defined by the differences in the values for the quality attributes of meat and carcass across the potential classification. Thus, a dendrogram exhibiting the possible clusters within turkey varieties was made using the underweighted pair-group method arithmetic averages (UPGMA) from the Universität Rovira i Virgili (URV), Tarragona, Spain and the phylogeny procedure of MEGA X 10.0.5 (Institute of Molecular Evolutionary Genetics, The Pennsylvania State University, State College, PA, United States).

#### 2.3.8 Discriminant function cross-validation

The leave-one-out cross-validation was used to validate the discriminant function used, aiming for at least 25% higher accuracy than that obtained by chance (118). The Press’ Q significance test was employed to compare the discriminating power using the following formula:


$${\mathrm{Press}}^{\prime }Q=\frac{{\left[N-\left(\mathrm{nK}\right)\right]}^{2}}{\left[N\left(K-1\right)\right]},$$

where *N* represents the number of observations of the sample; *n* represents the number of correctly classified observations; and *K* represents the number of groups (variety, in this case). To ensure Press’ *Q* statistic’s value is significantly better than chance, it was compared with the critical value of 6.64 for *χ*^2^ with one degree of freedom with a significance of 0.01 (118).

## 3 Results

### 3.1 Reliability of the canonical discriminant analysis model

No multicollinearity issues were reported in the preliminary analysis as all variables exhibited VIF values under 5. Hence, all variables were included in the further analysis of the present study.

Pillai’s trace criterion described significant differences in carcass and meat quality-related traits across turkey varieties and strains (*p* < 0.05). The values of the Pillai’s trace criterion and the Wilks’ lambda test are shown in Table 2.

**Table 2:** Summary of the results of Wilks’ lambda test and Pillai’s trace of equality of covariance matrices of canonical discriminant functions.

Wilks’ lambda		Pillai’s trace	
Lambda	0.0092	Trace	3.2717
F (observed value)	5.9949	F (observed value)	4.3533
F (critical value)	1.0890	F (critical value)	1.0885
DF1	744	DF1	744
DF2	15,938	DF2	20,520
Value of *p*	<0.0001	Value of *p*	<0.0001
Alpha	0.05	Alpha	0.05

### 3.2 Canonical coefficients, loading interpretation, and spatial representation

A total of 24 discriminating canonical functions compounded the discriminant analysis. The first eight functions (F1, F2, F3, F4, F5. F6, F7, and F8) were significantly discriminant and contributed 94.48% to the whole variance explanation, as shown in Table 3. By contrast, the rest of the functions were not significantly discriminant (*p* < 0.05).

**Table 3:** Eigenvalues and Bartlett’s test for eigenvalue significance.

	Eigenvalue	Bartlett’s statistic	Value of *p*	Discrimination (%)	Cumulative %
F1	2.9839	4024.1026	0.0000	382.092	38.2092
F2	2.3878	2838.1171	0.0000	305.752	68.7845
F3	0.6765	1791.2140	0.0000	8.6632	77.4477
F4	0.4948	1347.8543	0.0000	6.3365	83.7842
F5	0.3016	1002.9195	0.0000	3.8624	87.6466
F6	0.2208	776.7357	0.0000	2.8270	90.4736
F7	0.1683	605.5772	0.0000	2.1547	92.6283
F8	0.1449	472.1361	0.0154	1.8552	94.4835

Meat and carcass quality-related traits were ranked according to their discriminant capacity through the test of equality of group means (Table 4). Positions in the rank were assigned. A lower value for Wilks’ lambda and a higher value of F indicate the greatest discriminant ability (118).

**Table 4:** The results for the tests of equality of group means to test for difference in the means across meat and carcass quality traits groups once non-significant variables have been removed.

Variable	Rank	Lambda	F	DF1	DF2	Value of *p*
Cold carcass weight	1	0.3035	63.3091	31	855	<0.0001
Slaughter weight	2	0.3745	46.0609	31	855	<0.0001
Muscle fiber diameter	3	0.7137	11.0633	31	855	<0.0001
Sex – Female	4	0.7841	7.5923	31	855	<0.0001
Carcass/piece weight	5	0.7870	7.4636	31	855	<0.0001
b* meat	6	0.8583	4.5535	31	855	<0.0001
Ash	7	0.8944	3.2570	31	855	<0.0001
pH24	8	0.9036	2.9412	31	855	<0.0001
L* meat	9	0.9061	2.8598	31	855	<0.0001
Moisture	10	0.9072	2.8223	31	855	<0.0001
Fat	11	0.9127	2.6373	31	855	<0.0001
Water-holding capacity	12	0.9277	2.1494	31	855	0.0003

**Figure 2:**
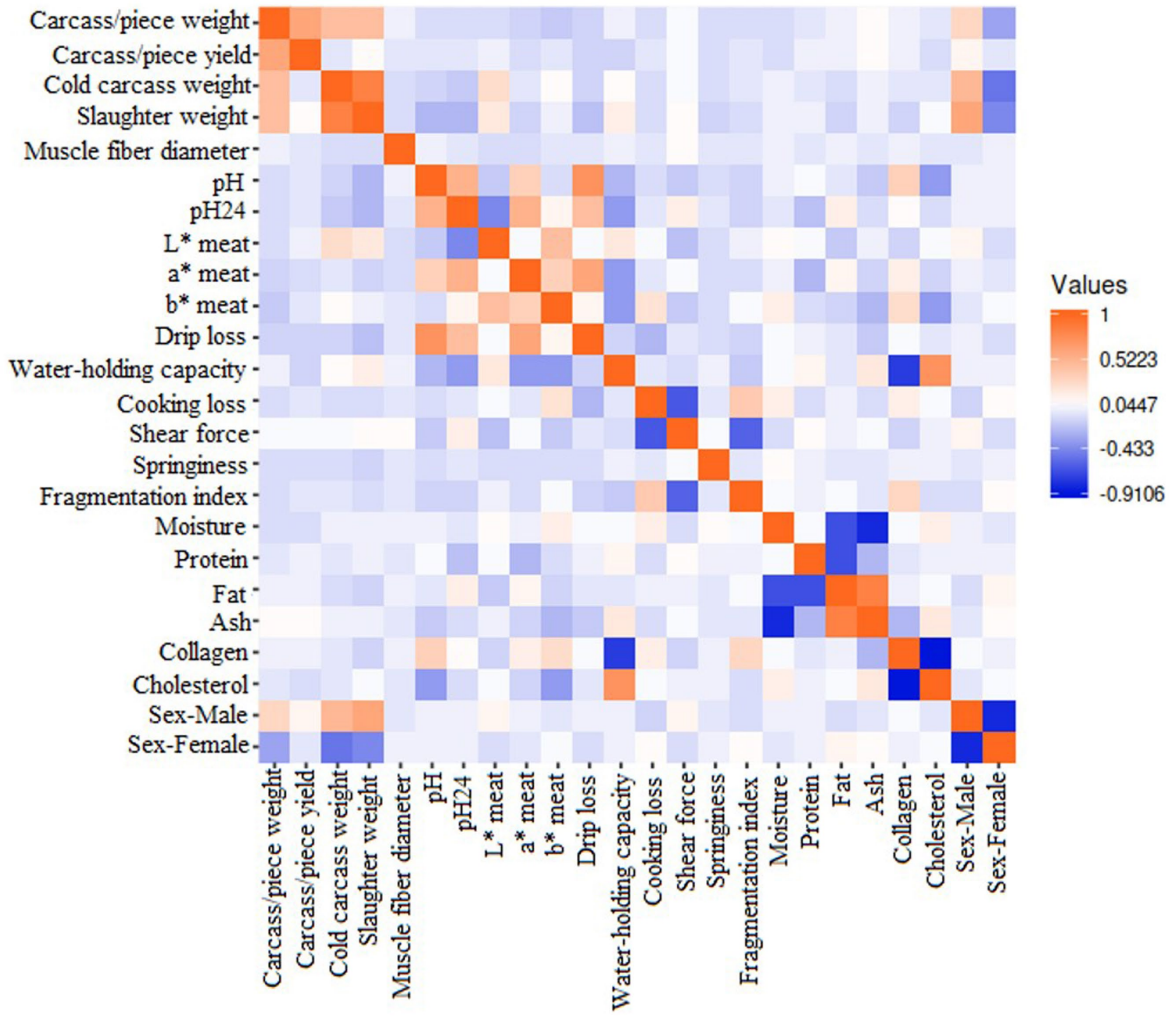
The correlation matrix between meat and carcass quality-related attributes included in the present study.

The correlation matrix values among meat and carcass attributes ranged from +0.595 to −0.654 and are represented in Figure 2.

**Figure 3:**
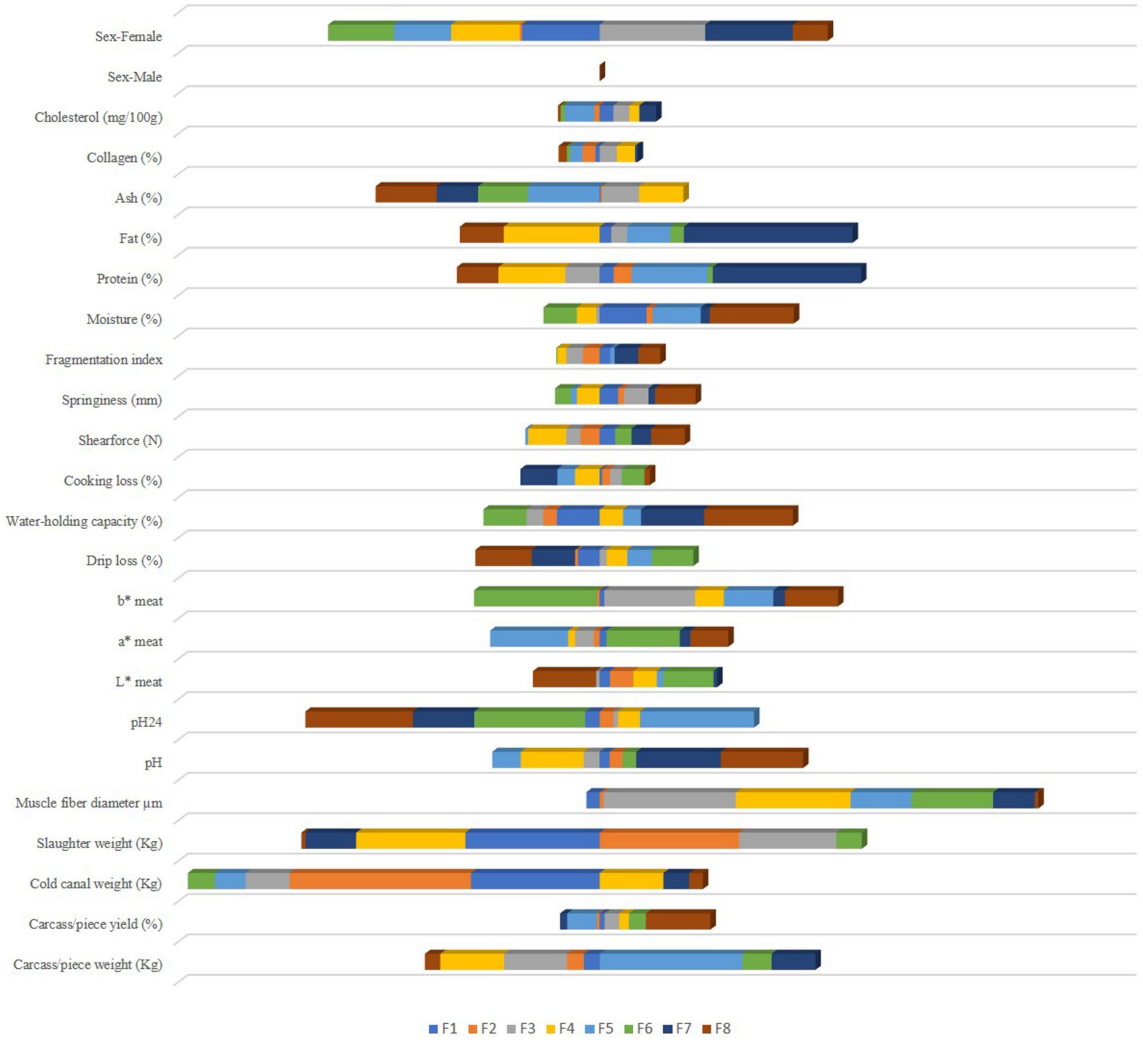
Discriminant coefficients for turkey meat and carcass quality attributes on each discriminant function.

Standardized discriminant coefficients are shown in Table 5. By evaluating these coefficients, possible reductions in the discriminant power of the variables can be detected as a consequence of multicollinearity between pairs. Moreover, the relative weight of each meat and carcass trait across the discriminant functions has been represented in Figure 3.

**Table 5:** Discriminant loadings for meat and carcass quality attributes.

	F1	F2	F3
Carcass/piece weight (kg)	−0.2914	−0.1376	−0.1984
Carcass/piece yield (%)	−0.0365	0.1020	−0.0546
Cold canal weight (kg)	−0.8123	−0.5223	−0.1139
Slaughter weight (kg)	−0.8498	0.2314	0.1401
Muscle fiber diameter (μm)	−0.0391	0.0076	0.5641
pH	0.0136	0.0287	−0.1558
pH24	0.0197	−0.0034	−0.0019
L* meat	−0.1326	−0.0152	0.0612
a* meat	−0.0042	−0.0166	0.0321
b* meat	−0.0451	0.0100	0.3706
Drip loss (%)	−0.0122	0.0051	−0.339
Water-holding capacity (%)	−0.1679	0.0079	−0.0531
Cooking loss (%)	0.0083	0.0252	0.1412
Shear force (N)	−0.0021	0.0037	−0.0655
Springiness (mm)	0.0605	0.0011	0.0683
Fragmentation index	0.0108	−0.0154	−0.0172
Moisture (%)	0.1189	0.0002	−0.0199
Protein (%)	0.0251	0.0396	−0.1626
Fat (%)	−0.0093	−0.0223	0.2068
Ash (%)	−0.0887	0.0169	0.1446
Collagen (%)	0.0467	−0.0344	0.0255
Cholesterol (mg/100 g)	−0.0121	0.0170	−0.0175
Sex-Male	−0.2826	0.3283	−0.4714
Sex-Female	−0.0714	0.3886	0.1470

**Figure 4:**
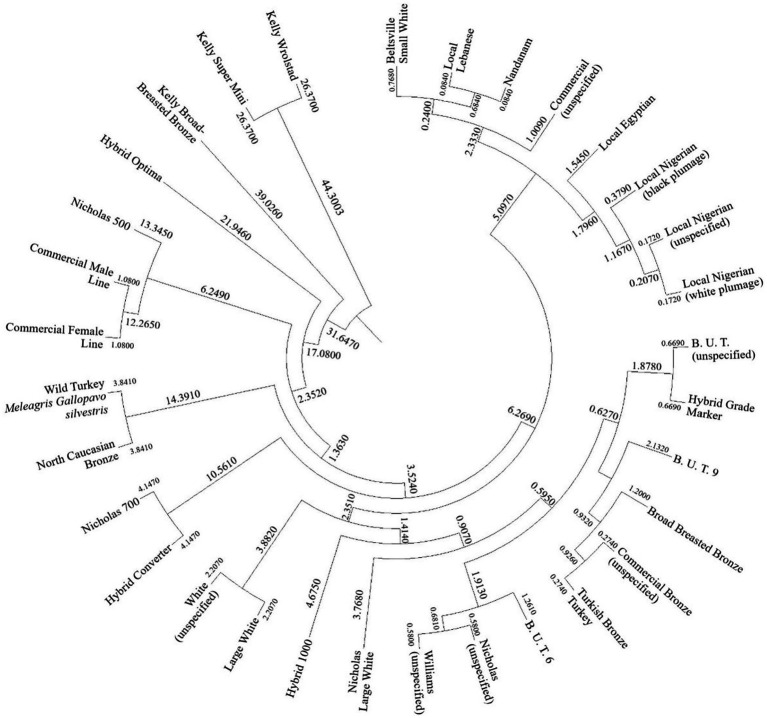
Cladogram constructed from the Mahalanobis distance between turkey strains and varieties.

The Mahalanobis distance were used to cluster turkey varieties and strains attending to carcass and meat quality traits. In this respect, the Mahalanobis distance represent the hit rate of matching an unknown observation into a particular classification group, which are turkey varieties, attending to its intrinsic carcass and meat characteristics. Hence, the likelihood of matching an observation into a group was estimated according to the literature (123). The Mahalanobis distance obtained after the evaluation of the discriminant analysis matrix were graphically expressed in a cladogram after their transference to squared Euclidean distance (Figure 4).

## 4 Discussion

### 4.1 Background

The domestication and migration processes have resulted in a great variety of livestock breeds (3), which have evolved and specialized to fit specific environments and human purposes (124). This is exemplified in the greater phenotypic diversity among breeds than that observed across wild populations (3). However, phenotypic variability between turkey breeds is much more limited compared with the fowl breeds (3), which could be attributed to a smaller effective turkey population size and a narrower effective population size before specialized strains appeared in the 20th century (124). Such specialization was translated into the development of a few highly selected strains used worldwide (125, 126), which, on the other hand, caused a reduction of the overall genetic diversity. This situation becomes even more worrying when considering the already low rates of diversity of the ‘heritage landraces’ that gave origin to these modern hybrids (3).

In addition to this limited phenotypic variability, a remarkable lack of literature addressing local breeds compared with industrial hybrids hinders the research of meat and carcass attributes. This disequilibrium is a direct consequence of the lack of resources allocated to the study of native genotypes (18), which in turn are mainly carried out in developing countries. It can be evidenced in the present study, as the only articles approaching local breeds were performed in Nigeria, Egypt, Lebanon, Bulgaria, and Turkey. However, meat and product characterization studies are essential for achieving the official recognition of a local breed (20), which is crucial for implementing further conservancy proceedings. Moreover, the knowledge of the attributes of their products allows the enhancement of their value by those consumers demanding for extra-quality and recognizable products (39).

### 4.2 Normality assumption and differences between turkey landraces and varieties

The Shapiro–Francia W’ test reported a few variables not showing statistical differences, which are as follows: pH, a* meat, b* meat, drip loss, cooking loss, shear force, and collagen. These attributes have previously reported controversial and inconsistent results in the literature comparing turkey genetic lines (4, 6, 8, 13). Thus, they could be suggested not to be approached in the breed traceability and differentiation of turkey meat products. Those traits are generally correlated, as pH directly influences meat color (4, 8, 30) and other textural traits such as drip loss (4, 8, 13, 30), cooking loss (4, 13), and shear force (4, 13). Those attributes might be expected to differ across turkey genotypes, as they are a consequence of the rate of pH decline (13) and muscle fiber diameter (8), which have been described as highly breed-dependent traits (4, 7, 8, 13, 30). However, the lack of variability found in the present study could be attributed to other influencing factors such as the rearing system (8), pre-slaughter wing flapping, or a different time of parameter measurement among studies (13). The controversial results are also found on meat collagen differences across strains. Some authors describe a lack of variability across two heavy commercial strains (6), while others found differences comparing slow- and fast-growing lines (30).

### 4.3 Meat and carcass quality-related traits correlation

The correlation matrix among variables depicted in Figure 2 describes a high positive correlation between carcass/piece weight to carcass/piece yield (+0.393), cold carcass weight (+0.266), and slaughter weight (+0.252). In this respect, factors influencing carcass/piece weights include slaughter age, rearing system, sex, and genotype (67, 127). Genotype plays an important role, as the exhaustive selection for meat production in specialized strains has been derived not only in the rise of body weight and growth but also in the enhancement of edible components (128), which is the consequence of the high value that certain parts of the carcass, especially the breasts, can achieve since most of the commercial turkey production is destined for further processing (127, 129). As the weight of a carcass or its cuts is employed in the estimation of its yielding (33, 106), those parameters were expected to be highly associated. The positive association between both cold and slaughter carcass weights with its components suggests a continuous development of body components as the animal grows. However, the yielding of the carcass or its cuts reported no correlations with the weight at slaughter or of the cold carcass. This finding could be contradictory when applying the allometric growth principles. Allometric growth holds great bone development at the hatching moment, which then leads to rapid muscle development and, in later stages of growth, to fat deposition (130). Hence, a greater yielding of muscular cuts (such as breasts and thighs) could be expected with increased live weight. This lack of association could be due to differences in the allometric growth pattern across breeds (127) or to a different age at slaughter between genotypes (67). In this respect, genotypes might differ in the time needed to reach slaughter weight or are subjected to different consumption preferences and cultural habits (131).

pH and pH24 showed a moderate positive correlation between them (+0.327), which has been previously reported in the literature (4). Muscle transformation into meat is caused by post-mortem anaerobic glycogen metabolism in the muscle (30), which causes tissue acidification. Hence, this process can be monitored by measuring pH at different stages post-mortem, usually shortly after the slaughter and a few hours later (4, 8, 13). This meat acidification process is particularly quick in poultry compared with other species, accounting for a few hours to its achievement (132). The positive correlation among these variables could suggest that, due to the speed of the process, pH could serve as an indication of the following meat acidification (measured as pH24). However, this association might not be linear, as pH did not show explanatory properties in the test of equality of groups, while pH24 did.

Both pH parameters showed a strong positive correlation with drip loss (pH: +0.490; pH24: +0.237), possibly due to the influence of the meat acidification process on water captivity attributes such as drip loss (8, 30, 47, 111). However, while the literature describes positive associations between drip loss and pH (4, 133), a negative correlation with pH24 is generally reported (4, 38, 101). This is because intense pH declines worsen water captivity attributes (4). Meat redness (a* meat) was also positively associated with acidification parameters (pH: +0.157; pH24: +0.301), as reported in the bibliography (7, 54, 75, 133, 134). In this respect, meat redness (a*) reflects the myoglobin content of the muscle fiber, which is directly associated with pH (101) and rapid, harmful pH decreases in meat due to quick glycolysis, resulting in redder meat (54).

The results obtained in the present study support the correlation among all colorimetry traits (L* meat-a* meat: +0.087; L* meat-b* meat: +0.316; a* meat -b* meat: +0.213), which has been previously reported by some authors (4, 8). However, the most widespread assumption holds that, when lightness (L*) increases, lower values of redness (a*) and greater yellowness (b*) could be expected (7, 36). Hence, no conclusive results could be achieved in this respect, as reported in the bibliography (7). This inconclusiveness could be due to the great variety of factors influencing colorimetry, such as genotype, diet, rearing system, season of slaughter, type of muscle, slaughter method, and time of measures post-mortem (6, 7, 13). These complex interactions between meat color traits highlight the need for further studies focused on their correlations.

Water-holding capacity showed negative correlations with a* meat (−0.157) and b* meat (−0.163). By contrast, b* meat showed positive associations with cooking loss (+0.149). This finding could be attached to the correlation of meat redness and yellowness to the pH decrease rate. Thus, the a*/b*ratio has been employed as an indicator of myoglobin oxidation, and a correlation with the rate of post-mortem fall was found (30). Rapid decreases in pH fall led to a more intense red and yellow color (54), which also could promote the denaturation of myofibrillar proteins, reducing water-holding capacity (36).

pH showed a moderate positive association with collagen (+0.194). Association among meat collagen and pH could be attached to the fact that both variables are influenced by characteristics of muscle fiber structure (37) and its connective tissue (6). However, a negative correlation between those traits might be expected according to described in the literature (73). Authors found meat collagen to be negatively correlated with pH and positively associated with muscle glycogen. Thus, fast-growing birds, which are usually early slaughtered, develop immature muscle collagen (6) and show lower meat acidification (higher meat pH) (4).

pH24 showed a moderate negative correlation with meat lightness (L* meat) (−0.223). This negative genetic correlation has been described before in turkey (54, 73), as well as in chicken (135, 136) and Japanese quail (38). pH24 reported a negative association with water-holding capacity (−0.172) as well, which was also described in chicken (136) and Japanese quail (38). Therefore, as pH24 increases (and meat lightness decreases), the water-holding capacity attributes of the meat are improved. This finding is reinforced by the slight positive correlation between lightness and WHC (+0.119) obtained in the present study. Hence, paler meat with a lower pH is usually associated with greater cooking losses and, hence, a reduced water-holding capacity (73).

The fragmentation index is moderately positively associated with cooking loss (+0.174). The fragmentation index is used as an indicator of meat textural attributes (137), describing post-mortem muscle protein degradation (138). This protein denaturation plays an important role in the cooking loss variable (137, 139), as denatured protein is less soluble than physiological protein (139), leading to greater humidity wastes during culinary processes (140). Furthermore, the fragmentation index showed a moderate positive association with meat collagen (+0.194), which is another meat textural indicator (13, 134, 141). In this respect, meat collagen has been described to influence the fragmentation index in woody-breast filets in chicken (142, 143).

Fat is strongly and positively associated with ash (+0.595). Regarding these traits, contradictory results are found in the bibliography. A positive association between traits has been described (144), and parallel tendencies of variables were reported when comparing turkey genotypes (8) and rearing systems (7). On the other hand, a negative correlation (144) and no correlation (145) among traits have been reported as well. This lack of consistency could be due to the great variability of factors influencing meat chemical composition, as the ash proportion is influenced by genotype, sex, age, rearing system, and nutrition (146). The aforementioned variables (fat and ash) showed negative correlations with moisture (−0.375 and −0.498, respectively), which is supported by literature (144, 147, 148), which could be attached to the fact that greater fatty muscles repulse moisture due to their low solubility (147). Fat was also found to be negatively associated with protein (−0.460), which is reinforced by previous authors (149). Moreover, muscle type (146) and rearing system (7) should be considered among those variables influencing meat composition. An inverse behavior has been described for these macronutrients in outdoor systems. Free-access systems are suggested to facilitate muscle (protein) development due to exercise and prevent fat deposition due to greater energy losses allocated to thermoregulation (7).

Cholesterol is positively associated with water-holding capacity (+0.434) and moisture (+0.189). However, the results showed no association between meat fat and cholesterol inclusion. This finding has been widely described before (150–152), as great cholesterol inclusion is not dependent on meat fat content. The low inclusion of cholesterol is a desirable trait in poultry, even though it serves as a great meat quality index (83). Caponization of birds seeks to increase meat lipids and cholesterol, which is translated into improved quality traits such as tenderness (153, 154). A possible explanation underlying this increase could be the important structural function cholesterol performs on cell membranes (155) and, hence, could prevent moisture losses from the destruction of cell walls (156). Moreover, descriptions of reduced moisture losses have been attributed to greater fat losses during cooking (157). Cholesterol also showed a strong negative correlation with collagen (−0.654). Meat collagen has been reported to mature with age (158), while cholesterol deposition is known to decrease (159).

### 4.4 Differences across turkey landraces and varieties

The results of the test of equality of groups show variables statistically explaining differences across turkey varieties. In the present study, we developed a tool to assess the improvement of meat differentiation and product traceability. A strong influence of weight-related traits is evidenced, particularly in cold carcass and slaughter weight variables. Due to the intense selection for growth that turkey species have experienced over the last few years (160), great phenotypic variability across breeds could be expected in live weight. Moreover, body weight is one of the most differentiating traits among breeds of poultry, which has been shown in unselected poultry breeds such as Brianzolo and Nero d’Italia turkeys (161) or Utrerana avian breed (15). On the other hand, live weight is a main classification criterion in the hybrid commercial industry (4, 7, 13).

Muscle fiber diameter showed strong explanatory properties as well, in parallel with the descriptions of a study on worldwide native chicken populations (18). Muscle fiber size varies among genotypes in poultry and other domestic species, as muscle growth is caused by an increase in fiber diameter instead of cell hyperplasia (8). The most widespread assumption among livestock species is that the fast-growing genotypes have larger muscle fibers than slow-growing lines (162). However, differences can be observed even within fast- and slow-growing strains (8).

The female sex resulted as a great differentiating variable, which might suggest a great variability across turkey breeds in the effect of sexual dimorphism on meat and carcass attributes. Turkey species show a great sexual dimorphism in morphometric traits (45), which is particularly evident for body weight traits. However, a different effect of sexual dimorphism on body weight is exhibited. For example, males from native genotypes tend to double or triple female body weight (17, 163), while this difference has been described to be lower in commercial turkeys (164).

Some of the technological properties (yellowness, lightness, pH24, and WHC) were also discriminating among turkey varieties. Those are traits mainly depending on muscle post-mortem metabolism, particularly the meat acidification process (30). Hence, differences among breeds and varieties on factors influencing muscle metabolism (such as glycogen fiber reserves, and pre-slaughter stress predisposition) could explain these results (4, 7, 13, 30). Meat nutritional traits (ash, moisture, fat) showed explanatory properties as well. However, inconsistent and no conclusive results are found in the literature considering breed differences in meat chemical composition (6–8, 30, 31).

### 4.5 Mahalanobis distance between landraces and varieties

Finally, the cladogram reflects the Mahalanobis distance performed by the discriminant analysis across turkey varieties. Interesting results are found attending to the clustering pattern. Some of the turkey landraces showed unspecified designations in their respective studies. However, they have been clustered close to each other, which is the case of the “female line” and “male line” nomenclature used (70), which were clustered close to the ‘Nicholas 500’ strain. A similar situation was found in the “White” strain, in Turkey (104), which was grouped near “Large White,” according to the developed discriminating tool.

Another interesting result is the clustering of bronze strains together (Broad Breasted Bronze, unspecified Commercial Bronze, and Turkish Bronze). This fact suggests their proximity. According to previous authors, this Turkish Bronze strain is widespread in Turkish rural areas and is reared under extensive systems. However, some authors also suggest that the origin of this breed in the American bronze. Therefore, its origin would be the same as the strain Broad Breasted Turkey (104).

Moreover, the cladogram clusters African breeds together. Additionally, within the Nigerian turkey, the unspecified feathered animals (107, 108) were clustered close to the white variety (106), which can be understood as those unspecified belonging to the white feather variety.

Finally, there was a final cluster with Beltsville Small White, Nandanam, and Local Lebanese. The Nandanam line descends from the Beltsville Small White breed, as this strain is obtained by crossing with the Indian “Desi” native breed (165), which could explain their similarities in carcass and meat characteristics, as they are grouped closely. Additionally, the “Local Lebanese” strain was clustered close to the Nandanam line, which evidences the closeness of these two genotypes. Due to the lack of literature on this landrace, a possible importation of Nandanam turkeys to Lebanon could be suggested (165).

## 5 Conclusion

In the present study, a DCA based on turkey meat and carcass quality attributes has been developed as a tool for addressing breed traceability trials. These trials lie in the description of highly differential traits across turkey strains and breeds, as well as the descriptions of strong associations among variables. Carcass weights and yields showed the greatest explanatory power, especially cold carcass weight and slaughter weight. Sex described good explanatory properties as well, possibly due to a different effect of sexual dimorphism across genotypes. Among other explanatory variables are some of the meat’s physical (colorimetry and pH traits) and nutritional quality traits. According to the correlations of attributes analyzed, strong positive and negative associations have been described between and within physical and nutritional traits. Thus, positive correlations were found among carcass yield variables, particularly those comprising carcass/piece weight. Muscle post-mortem metabolism produced strong associations between pH, colorimetry, water captivity, and meat chemical composition traits. Finally, the analysis of the Mahalanobis distance suggested the feasibility of this tool as a breed-clustering feature due to the aggrupation of known genetically close populations. Hence, the statistical tool developed in the present study could be employed in the post-mortem phase in turkey slaughterhouses, as it allows breed discrimination of both carcasses and cuts. This tool could be employed to protect breed-based products to avoid fraud or hybridization. Moreover, the selection of the most highly explanatory traits and those more representative in terms of greater correlations allows for the simplification and cost reduction of this tool, which can benefit those low-income smallholder farmers growing indigenous genotypes. However, contradictory results found in the bibliography and the lack of consensus among authors about some aspects highlight the need for further studies, especially for local breeds, which have not been deeply studied.

## Data availability statement

The original contributions presented in the study are included in the article, further inquiries can be directed to the corresponding authors.

## Author contributions

JS: Conceptualization, Investigation, Software, Writing – original draft, Writing – review & editing. AG: Conceptualization, Data curation, Formal analysis, Investigation, Methodology, Resources, Software, Supervision, Validation, Visualization, Writing – original draft, Writing – review & editing. FN: Conceptualization, Data curation, Formal analysis, Investigation, Methodology, Software, Supervision, Validation, Writing – review & editing. JL: Investigation, Resources, Software, Writing – review & editing. ED: Investigation, Writing – review & editing. JD: Project administration, Resources, Supervision, Visualization, Writing – review & editing. MC: Funding acquisition, Project administration, Resources, Supervision, Visualization, Writing – review & editing.

## Footnotes

^1^ www.heatmapper.ca
